# Where there is hope: a qualitative study examining patients’ adherence to multi-drug resistant tuberculosis treatment in Karakalpakstan, Uzbekistan

**DOI:** 10.1186/s12879-016-1723-8

**Published:** 2016-07-28

**Authors:** Shona Horter, Beverley Stringer, Jane Greig, Akhmet Amangeldiev, Mirzagaleb N. Tillashaikhov, Nargiza Parpieva, Zinaida Tigay, Philipp du Cros

**Affiliations:** 1 Médecins Sans Frontières (UK), Lower Ground Floor, Chancery Exchange, 10 Furnival Street, London, EC4A 1AB UK; 2 MSF, Nukus, Uzbekistan; 3 Ministry of Health of the Republic of Uzbekistan, Tashkent, Uzbekistan; 4 TB II Hospital, Nukus, Uzbekistan

**Keywords:** MDR-TB, Multi-drug resistant tuberculosis, Adherence, Qualitative study, Drugs, Autonomy, Side effects

## Abstract

**Background:**

Treatment for multi-drug resistant tuberculosis (MDR-TB) is lengthy, has severe side effects, and raises adherence challenges. In the Médecins Sans Frontières (MSF) and Ministry of Health (MoH) programme in Karakalpakstan, Uzbekistan, a region with a high burden of MDR-TB, patient loss from treatment (LFT) remains high despite adherence support strategies. While certain factors associated with LFT have been identified, there is limited understanding of why some patients are able to adhere to treatment while others are not. We conducted a qualitative study to explore patients’ experiences with MDR-TB treatment, with the aim of providing insight into the barriers and enablers to treatment-taking to inform future strategies of adherence support.

**Methods:**

Participants were purposively selected. Programme data were analysed to enable stratification of patients by adherence category, gender, and age. 52 in-depth interviews were conducted with MDR-TB patients (*n* = 35) and health practitioners (*n* = 12; MSF and MoH doctors, nurses, and counsellors), including five follow-up interviews. Interview notes, then transcripts, were analysed using coding to identify emerging patterns and themes. Manual analysis drew upon principles of grounded theory with constant comparison of codes and categories within and between cases to actively seek discrepancies and generate concepts from participant accounts. Ethics approval was received from the MoH of the Republic of Uzbekistan Ethics Committee and MSF Ethics Review Board.

**Results:**

Several factors influenced adherence. Hope and high quality knowledge supported adherence; autonomy and control enabled optimal engagement with treatment-taking; and perceptions of the body, self, treatment, and disease influenced drug tolerance. As far as we are aware, the influence of patient autonomy and control on MDR-TB treatment-taking has not previously been described. In particular, the autonomy of married women around treatment-taking was potentially undermined through their societal position as daughter-in-law, compromising their ability to adhere to treatment. Patients’ engagement with and adherence to treatment could be hindered by hierarchical practitioner-patient relationships that displaced authority, ownership, and responsibility from the patient.

**Conclusions:**

Our findings reinforce the need for an individualised and holistic approach to adherence support with engagement of patients as active participants in their care who feel ownership and responsibility for their treatment.

## Background

In 2013, approximately 480,000 people globally developed multi-drug resistant tuberculosis (MDR-TB), defined as TB resistant to rifampicin and isoniazid [1]. Treatment for MDR-TB is lengthy, has low efficacy, often causes severe side effects, and raises adherence challenges [2]. Adherence to medical treatment is a complex behavioural issue, influenced by many factors that are often context specific [3]. In MDR-TB treatment, poor adherence can have medical and public health implications [4–7].

A meta-analysis of MDR-TB treatment studies across 31 countries found a pooled proportion of patients lost from treatment (LFT; patients whose treatment is interrupted for at least two consecutive months) of 14.8 %. Strategies associated with reduced LFT include engagement of community health workers in administering and observing treatment, smaller cohort sizes through decentralised care, patient education, and a package of adherence interventions such as nutrition and financial support [5]. It is possible that some patients classified as LFT are actually treatment failures [8, 9]. The most frequently cited factor associated with LFT is alcohol use [4, 5, 7, 8, 10–13], others being a history of imprisonment [6, 8, 12], more severe drug resistance [8, 14], and socio-demographic factors including homelessness and lower education levels [12, 14]. Some data are contradictory, for example both employment and unemployment have been associated with LFT [7, 12], as have the intensive and continuation phases of treatment [15, 16]. Side effects, fear that treatment is ineffective or harmful, and perceptions around ill-health and cure can also lead to patients not completing treatment [8].

Uzbekistan is one of the high priority countries for drug-resistant TB in the European region, with 23 % MDR TB amongst new TB cases [1]. In Karakalpakstan, an autonomous region in the northwest of the country, proportions of MDR-TB were over 40 % of new and over 75 % of previously treated cases in 2010–2011 [17], which indicated the presence of primary transmission as well as resistance amplification. Médecins Sans Frontières (MSF) and the Ministry of Health (MoH) operate a TB programme in accordance with international guidelines in Karakalpakstan, where the model of treatment and care is decentralised, and ambulatory from day one [18]. Daily MDR-TB treatment is administered directly observed (directly observed treatment; DOT) at “DOT corners” within ambulatory health facilities, or at patient homes for certain patients with challenges accessing their nearest health facility.

Among patients starting treatment for MDR-TB between 2009 and 2012 (*n* = 1,190), 57 % had a successful treatment outcome, 27 % were LFT, 9 % died, and 7 % failed treatment. Adherence support strategies in the programme include: individual pre-treatment assessment by counsellors to provide information and assess readiness for starting treatment; one-to-one counselling or psychiatric support if needed; engagement of family members and family information sessions (with patient consent); peer support group meetings; and social support. In addition, health practitioners receive training sessions on adherence and communication. However, rates of LFT from MDR-TB treatment remain high.

While certain factors associated with LFT have been identified, there is limited understanding of why these factors interfere with completion of treatment, and why some patients are able to adhere to treatment while others are not. We therefore conducted a qualitative study to determine the reasons for adherence and non-adherence in MDR-TB patients in Karakalpakstan, Uzbekistan. Our aim was to provide insight into the patterns of patients’ adherence, exploring the barriers and enablers to treatment-taking in order to inform future strategies of adherence support.

## Methods

A qualitative study design was adopted in order to examine patient experiences with MDR-TB and treatment-taking, including the challenges and supportive factors, from their perspectives, as well as from the perspective of health practitioners. This study was conducted towards the end of 2014 and included in-depth interviews with MDR-TB patients and health practitioners.

### Sampling criteria

Patient participants were identified via purposive sampling in order to recruit those best able to provide insight into adherence to MDR-TB treatment, and to achieve maximum variation in treatment-taking experiences. Health practitioner participants were also included in order to explore their experiences working with MDR-TB patients and their views and observations regarding the barriers patients face and enablers that help them overcome challenges. In addition, inclusion of health practitioners was intended to allow further depth of insight into the treatment-taking process and staff-patient relationships.

Follow-up interviews were conducted with selected participants to enable interpretive theories generated from emerging data to be further examined [19]. Participants for follow-up interview were identified through reviewing interview transcripts and considering areas where depth of insight could be expanded.

### Sampling frame

Patients’ treatment-taking information was obtained from the project database, which included health practitioner DOT records of doses prescribed versus those observed as being taken (for example, where patients do not present to the clinic for daily observed treatment, or refuse certain drugs). Patients were stratified by adherence into three categories: adhered well (observed taking over 90 % of treatment as prescribed); partially adhered (observed taking up to 50 % of treatment as prescribed); and LFT within the preceding 6 months. Sample recruitment was also stratified for patient characteristics, including gender and age. Health practitioners were identified to include MSF and MoH practitioners in a range of professions including doctors, nurses, and counsellors, who all had experience supporting and/or treating patients with MDR-TB.

### Participant recruitment

Patient participants were contacted by members of the psychosocial team with information about the study asking whether they would be willing to be interviewed. Interviews were arranged with those who agreed to take part at a time and location convenient for them, which was either at their home or in a private room at the DOT corner (outpatient TB department). Health practitioners were given information about the study by the principal investigator and asked if they would be willing to take part, with interviews then held in a private room of the project office or DOT corner.

### Data collection

In-depth interviews were conducted from September to November 2014. All interviews (*n* = 52) were carried out by the principal investigator, the majority in the local language (Karakalpak) with a trained interpreter, or in English. Interviews were participant-led and encouraged natural flow of conversation, based on topic guides to explore specific areas. Interviews lasted between 30 and 90 min, with an average duration of 60 min. Most interviews were audio recorded, unless participants refused consent for recording (*n* = 3), and were transcribed verbatim by the principal investigator if conducted in English (*n* = 4) or by an interpreter using equivalent translation from Karakalpak to English (*n* = 45), in order to maintain the integrity and meaning of data. Of the interviews transcribed by an interpreter, close to 50 % of these were transcribed and translated by an independent interpreter to maximise data validity. The three interviews that were not audio recorded were transcribed using notes. Topic guides for follow-up interviews were devised following review of initial interview transcripts and identification of areas to further explore. Data collection continued until new information was not being generated, therefore evidencing data saturation [20, 21].

### Data analysis

Data analysis began from the point of data collection and followed an iterative process. Thinking and theorising began with interview notes followed by interview transcripts, which were read and re-read, using coding to identify emerging patterns and themes. Analysis was performed manually, and drew upon principles of grounded theory [22, 23], with constant comparison of codes within and between cases to raise codes to a conceptual level and to generate theory. Analytic memos were used to track the development of analytic thought. A coding framework was developed and then transcripts were re-read to actively seek and explore discrepancies from majority codes and categories, as well as contradictory or unexpected findings, and to ensure findings presented were a true reflection of participant accounts. A second researcher reviewed the initial codes to enhance validity and minimise the effects of potential researcher biases.

## Results

52 interviews were conducted (35 MDR-TB patients, 12 health practitioners), including five follow-up interviews (four patients, one health practitioner). Patient participants were aged from 16 to 69 years, 54 % were female and 34 % had no prior treatment history. They comprised 15 ‘adherent’, 12 ‘partially adherent’, and eight LFT patients (Table 1). Health practitioner participants included five counsellors, five TB nurses, and two TB doctors (Table 2). Twenty-four percent of the 46 patients identified for recruitment did not participate for reasons including: being untraceable, e.g. patients who were abroad and therefore could not be invited to take part (*n* = 6); unavailable during the study timeframe (*n* = 2); fearing being brought back to treatment (one LFT patient); or unknown (*n* = 2). The majority of those who declined participation (*n* = 7) were LFT patients.

**Table 1:** Patient participant characteristics

Patient code	Gender	Age group (years)	Treatment-taking profile at time of interview	Treatment history
P01	Female	20–29	Partially adherent	New pt (no prior tx history)
P02 & P02 FU	Female	20–29	LFT	Extensive (DS to DR)
P03 & P03 FU	Female	20–29	Partially adherent	Extensive (DS to DR)
P04	Male	20–29	Adherent	New pt (no prior tx history)
P05	Male	40–49	Adherent	Tx after LFT from SCR
P06	Male	20–29	Partially adherent	Previous incomplete DS tx
P07	Female	20–29	LFT	Previous incomplete DS tx
P08	Male	50–59	Partially adherent	Previous incomplete DS tx
P09	Female	20–29	Partially adherent	Previous incomplete DS tx
P10	Male	20–29	Adherent	New pt (no prior tx history)
P11	Female	20–29	Adherent	Relapse
P12	Female	60–69	Adherent	New pt (no prior tx history)
P13	Female	20–29	Adherent	New pt (no prior tx history)
P14	Female	20–29	Partially adherent	Relapse
P15	Male	30–39	Adherent	New pt (no prior tx history)
P16	Male	20–29	Partially adherent	New pt (no prior tx history)
P17	Male	40–49	Adherent	Extensive (DS to DR)
P18 & P18 FU	Female	20–29	Adherent	Extensive (DS to DR)
P19	Male	16–20	Partially adherent	New pt (no prior tx history)
P20	Male	20–29	Adherent	Relapse
P21	Male	20–29	Adherent	Relapse
P22	Male	20–29	Adherent	New pt (no prior tx history)
P23 & P23 FU	Male	16–20	Adherent	New pt (no prior tx history)
P24	Female	30–39	LFT	1 previous course (failure after amplification)
P25	Female	20–29	LFT	New pt (no prior tx history)
P26	Female	60–69	LFT	1 previous course (failure after amplification)
P27	Female	60–69	LFT	1 previous course
P28	Male	20–29	Partially adherent	Relapse
P29	Male	30–39	LFT	Relapse
P30	Female	30–39	Partially adherent	Unknown
P31	Female	20–29	Partially adherent	Unknown
P32	Male	30–39	LFT	2 previous courses
P33	Female	30–39	Partially adherent	New pt (no prior tx history)
P34	Female	30–39	Adherent	1 previous course
P35	Female	30–39	Adherent	2 previous courses

*FU* follow-up interview, *Tx* treatment, *Pt* patient, *LFT* lost from treatment, *DS* drug sensitive, *DR* drug resistant, *SCR* short-course regimen (9 month MDR-TB treatment regimen being piloted)

**Table 2:** Health practitioner participant characteristics

HP position	Number of participants	Number of interviews
Counsellor	5	5
Nurse	5	6 (including 1 FU)
Doctor	2	2

*HP* health practitioner, *FU* follow-up interview

N.B. health practitioner positions have not been given for each participant code to protect confidentiality of participants, due to the small number of health practitioners for certain positions

Three main, interdependent themes were identified through data analysis as underlying patients’ treatment-taking experiences: 1. hope and better knowledge offering the potential to overcome non-adherence; 2. patient autonomy and control optimising engagement with treatment; and 3. perceptions of the body, self, treatment, and disease on tolerance. These themes and the framework comprising their sub-categories are described below, and are also displayed in Table 3.

**Table 3:** Coding framework after reduction of codes and categories

Theme	Supporting treatment-taking	Undermining treatment-taking
1. Hope and quality knowledge	-Acceptance of diagnosis -Perceived need for Tx -Belief in Tx efficacy -Hope for the future (perceived attainability of cure) -Peer-to-peer information -Active information seeking -Support and encouragement (counsellor, peer, family)	-Doubt, disbelief, shock, denial -Inadequate information and understanding -Restrictive HP advice -Distrust of health services -Myths and misinformation -Negative peer influence
2. Patient autonomy and control	-Ownership and self-responsibility -Valuing health -Strength of character, perseverance, motivation -HP support and encouragement	-Conflicting priorities -Sacrifices Tx requires -Authoritative practitioner-patient relationships -Gender roles -Stigma, shame, blame, isolation -Social/financial responsibility
3. Perceptions of self, body, Tx and disease on Tx tolerance	-Coping mechanisms -Visualisation techniques -Distractions from Tx -Mind set and positivity	-Drug side effects -Intolerance -Drug toxicity, poisoning -Tx fatigue

*Tx* treatment, *HP* health practitioner

### Where there is hope but limited knowledge

This theme explores the presence or absence of quality TB knowledge, seen to influence the diagnostic and treatment-taking process through impacting the views and beliefs of patients on their need for treatment and its perceived efficacy.

#### Limited TB knowledge and information

Most patients described limited TB knowledge and understanding, as well as not being aware of the disease prior to diagnosis, as relevant to adherence. Certain primary health practitioners also described insufficient TB knowledge among TB practitioners:

*I had no idea about lungs. I have not even heard of it. I learnt it when I got sick*. P04

Several participants, patients and health practitioners, described limited general health and TB knowledge in the wider community. Knowledge that did exist was described as being restricted to fear-driven messages such as TB having no cure, fears of infection, and TB developing as a perceived result of a curse or jinx:

*[Some] people in the community, the only thing they know is that it will infect, that’s all, they don’t think that it is curable*. P03 (follow-up).

*Locals say that it may be because of hex (jinx)… Actually there is hex in medicine. It may affect you lungs, head or eyes. Yes. Yes. It’s like a man loses his aura*. P26

The vast majority of patients described their MDR-TB developing through factors relating to “cold”, in terms of exposure to cold weather, wet ground, drinking cold water, or getting a cold. Many patients also understood their disease as having developed through not having looked after themselves, over-working, or through their immune system weakening, for example due to inadequate diet, giving birth, or physical trauma such as a car accident, which could lead to self-blame. For some participants, medical advice reportedly influenced these perceptions of disease development. A minority of participants believed they were infected with TB, or described their treatment history and drug resistance developing. Most patients asserted that they were not infected.

*It was cold in the house, I was feeling cold, couldn’t look properly after myself. And mostly the field work… I mean in 40 days after delivery women usually do not go outside and it was not like this with me, I had to…do the work…* P03

*They come and they say to put that on [mask]… I say that I don’t transmit this disease. Because I was not infected, I don’t know where I got it from. Otherwise none of our generations have had this, neither my own relatives nor here.* P27

Medical education or advice was described by some patients as restrictive for their health and wellbeing during and following treatment completion, including activities that must be avoided after treatment completion to avoid disease relapse, which contributed to patients’ fears about the permanence of cure.

*[Medical staff] they say to women not to give birth for 5 years. They say that during the delivery the lung may dislocate, if there is a wound it may opened again, and it may start bleeding.* P03

*They told us, they said “don’t lift anything more than 5 kg”, we submit to the discipline.* P22

The majority of participants described TB transmission as occurring through materials or objects. Some described it as being hereditary and only a few described airborne transmission. Health practitioners also mentioned the belief that TB is transmitted through materials or objects existing among certain TB health staff.

*You should keep yourself clean. You should keep your dishes separate… I have separate towel.* P26

*They [doctor] may be sitting with a TB patient in one room, but he sits like separating his dishes and avoiding touching that patient, but they are not afraid of sitting in one room [without respirator].* HP 11

The information provided to and understood by patients during the diagnostic process often appeared to be inadequate, with many patients describing their diagnosis having been explained as a spot or symptom in their lung(s), without the opportunity to develop a better understanding. Patients reported receiving a brief explanation of their disease by doctors, with counsellors being relied upon for more expansive information.

*I came and had X-ray they said “you have a spot in your lung, you will go to TB2 [a hospital]”… They didn’t say anything.* P12

*They said “your results are good but it is multi drug resistant type” and I asked what it was, they said that there was psychologist and we should see her.* P18

During their diagnosis certain patients described being in a state of shock in which it was difficult to process the information given to them; this was echoed by most health practitioners.

*When they told me I didn’t understand. They told me and I was so much in shock that I even don’t remember what I was thinking about that time*. P01

*If it is the first time that they were told that they have TB, even the health education part it’s too shocking… It seems like they were under a shocking period to accept the diagnosis even.* HP 01

Several patients had taken an active approach to information seeking relating to their disease and treatment, involving using the internet, reading books, and asking their peers or others. This was also described by some health practitioners.

*I started searching and I found that yes, this disease really exists, that this disease is really curable, that these drugs were not invented today nor yesterday, that they are being used for a long time, they use these drugs for more than 30–40 years.* P01

Patient-to-patient information was seen to have more credibility in terms of quality, trust, and belief in the information source. Those who had passed through treatment themselves were perceived by patients as knowing its reality and assumed more likely to be honest and reliable. However, there could be the risk of negative peer influence, for example through LFT patients who appeared well despite not completing their treatment course.

*If it is said by a person who takes the treatment it will be accepted… Because he knows how you used to suffer that’s why you believe him more than a healthy one*. P01

*I know many patients who’d been given a “default” outcome, but now they are good…some of them have even started a job*. P33

#### Quality information, knowledge, and belief supporting adherence

Several participants mentioned the significance of disease knowledge and understanding for acceptance and belief in treatment-taking. Some patients asserted that belief in the treatment efficacy and its impact on cure was the most important factor supporting the treatment-taking process. Most patients saw belief as being integral to the long and difficult treatment, giving strength to overcome its challenges.

*If they don’t have information, they won’t take treatment.* HP 07

*If you believe in it then you will be cured.* P21

Belief in treatment effect was reinforced through seeing symptoms and results improve, as well as seeing other patients recover. This instilled a sense of motivation and determination.

*Day after day I take the drugs, months are passing, my cultures are good, turning to negative, every 6 months I am being x-rayed, everything is good. And because I see such results I believe… I believed that I can be cured with these drugs*. P13

#### Doubt, myths, and adherence obstacles

Where patients doubted medical advice, including their diagnosis, and did not ascribe value to MDR-TB treatment, they appeared to be more vulnerable to misinformation, myths, and seeking alternative treatment. Doubt and distrust were reportedly exacerbated through changes in required treatment length and delays with accurate diagnosis (including awaiting drug sensitivity test results).

*The idea that they were telling me a lie was inside of me like a worm… I was like eating myself from inside not believing it* P01

*They told me that it would be extended until September and after that I started hating it completely and I had missed about 20 days because of that. I lost my inspiration to take the drugs.* P19

Several patients and health practitioners described myths, such as treatment being provided as part of a foreign drug experiment in which drug efficacy was not known and was being tested. This was said to instil fear in some patients and deter them from taking treatment, and could be seen as demonstrative of patients’ distrust in the health system.

*In the streets people tell different information that there are some trials are being held in our region.* P01

Certain patients appeared to be conflicted about trusting medical advice but also wanting to find an easier option or believing myths and misinformation. This led to consideration of alternative treatment options, seen to be less lengthy and arduous, and was also reportedly fuelled through limited health knowledge and understanding.

*Unless the doctor delivers him the information fully and explains in detail, that patient begins to look for that information from other people*. HP 11

*So this time we also came home and I took dried apricots. And I felt a bit better. After that I was living normal, thinking that I was cured.* P27

Some patients (in particular those LFT) did not believe they currently or had ever had TB. This was especially the case in asymptomatic patients, who expressed confusion linked to the lack of pain receptors in the lung. Disease denial and disbelief were seen as negatively impacting on treatment-taking.

*I still don’t believe I have had TB… they said I got this disease. We did not believe. It didn’t hurt anywhere.* P27 [LFT patient]

Patients’ understanding and belief also influenced decision-making about when cure had been achieved. Several patients believed they had been cured, for example once their symptoms and/or results improved, and therefore stopped treatment before completing the full course, including many of the LFT participants.

*I took the drugs for about 6–7 months, you know, then my coughing stopped, I put on weight and then my mother-in-law said “you look good, you don’t look like you are sick, you don’t have to take the treatment”.* P03 follow up [history of previous LFT]

*I said to myself I’m good, I won’t take the drugs for the rest of the time because I feel better. All the results were negative and good, and since I was also feeling good the rest was depending on me.* P24 [LFT]

#### Support and encouragement for treatment-taking

Support and encouragement from family, counsellors, and peers were seen to help channel patients’ strength, motivation, and determination in continuing with treatment. Most patients cited their counsellor relationship as supporting treatment-taking.

*She really helped me much in continuing the treatment*. P01

*I used to take treatment, take treatment well if I had a support from someone*. P02

Peer support was seen as providing a sense of shared experience and solidarity. Certain patients described forming their own peer support networks. For example, one patient had connected with other young MDR-TB patients, and they used social media daily to urge one another to take their treatment.

*I talk to them [other patients] through the Agent (Russian social network), and you ask them at that time like “have you taken your drugs”, you give advice like “don’t miss”, then you meet often, that’s why we are taking the drugs.* P23 follow-up

The need for more education and information about TB was highlighted by patients and health practitioners, at both the patient and community level. Suggestions were made for the use of a variety of mechanisms for information sharing, including interactive and multi-media based mediums such as film, radio, and television.

*Put a screen here, and we all sit together there and watch a short video, maybe for 10 min. And this video should show the patient with this disease and his way of treatment from the very beginning till the full completion, but do it in a very nice way. So that people who see it can believe in it.* P13

### Patients’ autonomy and control over their treatment

The second emerging theme explores positive engagement with the treatment-taking process as reliant on patients’ sense of autonomy and ability to prioritise treatment over and above conflicting demands on their daily lives. This can be challenged by common beliefs, myths, and stigma about TB, as well as authoritative practitioner-patient relationships.

#### Social and personal spheres influencing patient autonomy

Treatment for MDR-TB was seen to have significant impact on daily life, requiring sacrifices relating to education, employment, and duties and responsibilities such as housework, as well as having social implications. Many patients said that they had worked prior to starting treatment, but were no longer able to do so. Patients described feeling so weak after taking the drugs that all they could do was to lie down; they couldn’t concentrate on studying and felt unable to care for their children or perform duties around the home.

*I used to think of everything, like: “I am not able to do anything anymore”. I used to cry because of that. I was also thinking that I couldn’t look after my children. When I go home… I couldn’t do anything at home, couldn’t do anything.* P11

Single patients described feeling unable to marry until they had finished treatment, and several younger patients felt isolated from their friends and that they were missing out on activities that their peers were taking part in, such as attending events.

*I was young at that time, around 16. Young heart, you know. And 2 years, oh, wow… you have to be separated from your friends and so on for 2 years. So I thought about that, about 2 years of separation.* P19

Significant stigma was described by many participants, both perceived and enacted. Patients felt ashamed, as though people blamed them and looked at them differently because of their disease. They also described feeling ostracised and asserted wanting to hide their disease status.

*I felt like healthy people were looking at me with disgust*. P13

Many patients felt defined by their disease, their lives consumed by the routine of treatment-taking and side effects, separating them from a sense of normality and society. Some expressed a desire to feel equal, with a life beyond treatment and disease.

*That constant condition of half a man, I used to feel like a half man.* P01

*Actually there is nothing worse than staying at home and just take the drugs. I wish to complete this treatment, get cured, work like others, get salary, and attend celebrations and other events (crying).* P34

Gender and societal status were important determinants of patients’ autonomy and their ability to prioritise treatment. Most female patients who were, or had been, married described the implications of their role as daughter-in-law for treatment-taking. Many were expected to serve their husband’s family following marriage, with their position resulting in a lack of control over their health or lives. Some had severe difficulties continuing with treatment, including who described being seen as useless because side effects prevented their ability to perform household duties. Some patients were therefore unable to continue their treatment and only able to resume following divorce or leaving their husband’s family home, which was described as very difficult in the social context. Some female patients suggested that it could be better for daughter-in-law patients in this position to take treatment in hospital.

*While taking the treatment, my husband’s house were against… [they] wouldn’t let me take treatment… I took the drugs for about 6 months and then left, being a defaulter… What they say is: “we need someone who will serve us…”* P03

There were some counter examples, with some married women asserting that they received support from their husband and in-laws. These patients seemed to live in separate homes from their in-laws, and in certain cases their family members had received treatment education through engagement with counsellors, described as particularly helpful in achieving their husband’s support.

Many male patients expressed frustration at not being able to work while on treatment, some highlighted their responsibility to provide for their family, with one LFT patient explaining that this was why he felt no choice but to stop treatment early. Socio-economic barriers to treatment-taking were reiterated by health practitioner participants.

*Due to my family reasons I couldn’t… I had to stop the treatment. Because there was no other source of income.* P32 (LFT)

Age affected treatment-taking experience. Younger patients were perceived as being able to recover more quickly and better tolerate the drugs, whereas older patients expressed fatigue and found drug tolerance difficult, particularly if there were co-morbidities. Some older patients viewed themselves as having limited time left alive, with 2 years of treatment therefore being a significant burden. For certain younger patients, however, 2 years seemed a comparatively short period.

*Recovery will be quick… my organism is young*. P19

*Sometimes I don’t feel like taking that drug, you know. When I don’t feel like taking the drug, I say “I’ll stop from today on, I am 70 years old, that’s it, if I die it’s fine”.* P12

Patients who described valuing their health and who were able to prioritise it above other demands demonstrated ownership, autonomy, and self-responsibility for the treatment-taking process, which drove them to persevere. These patients accounted strength of character and motivation as relevant to treatment-taking, alongside having aspirations and plans for their lives post-treatment, for example having a family, getting married, working, or studying. Strength of character was also acknowledged by health practitioner participants as important in overcoming challenges with treatment; some associated this with family background and education level.

*I have my natural strength… I force myself every day, otherwise I won’t be able to take them. I can’t take them if I don’t push myself to it.* P27

*Every man decides for himself… I need my health for my own sake. Because I need it for myself I have decided it*. P21

*There are patients who become a defaulter because of social problems they have but there are also patients with social problems who are still trying to find a way out and to continue with the treatment to the end*. HP 09

#### The practitioner-patient relationship undermining patient authority

The power inherent in the practitioner-patient relationship was alluded to, with conflicting views about health practitioner communication style and approach. Certain patients described their relationship with doctors and nurses as positive, involving help and encouragement. Most of these patients seemed to be adherent, with some saying doctors praised them for their treatment-taking. Other patients highlighted the distrust that could exist within the relationship, and expressed being abused and threatened by doctors, and feeling angry about or afraid of practitioner attitudes. Some doctors were seen as just inspecting their treatment-taking, rather than performing full medical examinations and providing explanations. Patients described doctors begging them to take their drugs and feeling annoyed with them if they didn’t.

*I used to have fear of their attitude*. P13

*The doctors also get angry and they begged saying “take your drugs, take your drugs”… the doctors are also fed up with me.* P14

Health practitioner participants highlighted the need for improved staff-patient communication, trust, mutual commitment, and respect. Practitioners explained that the staff-patient relationship impacted treatment adherence. A relationship that centred on blame and inspection of drug taking was seen to hinder patients’ ability to share adherence challenges. Begging patients to continue with treatment was seen to undermine their autonomy and self-responsibility for treatment-taking.

*The communication level should be very improved, patients should be seen as people… he should be a participator in his treatment… give space for patient to decide in his situation rather than one sided communication.* HP 01

### Side effects from drugs and solutions for tolerance

How patients experience and perceive the impact of TB and treatment on their body presents the third and final theme. Such perceptions influence treatment tolerance and therefore the ability to continue to take treatment.

All patients described experiencing side effects at some point during treatment. A range of physical side effects were outlined, with many patients experiencing nausea and vomiting, loss of appetite, weakness, headaches, joint swelling, and pain, particularly in the legs. Patients stated experiencing dizziness and feeling drunk, changes in their appearance through skin discolouration, blurred vision, burning, itching, and diarrhoea. Psychological side effects were also described, including low mood, nervousness, loss of memory, and thinking negative, dark thoughts.

*I used to vomit until the evening… I used to feel dizzy, was almost about to fall down, felt so weak. Shivering… my hands and legs were shivering after vomiting.* P25

*I take the drugs. Then I am knocked down… mouth full of saliva, feel drunk, don’t have any power to do anything*. P27

Many patients viewed side effects as being worse at the beginning of treatment, reportedly becoming more tolerable after 2 to 3 months, when the body was said to adjust to the drugs. However, there were two exceptions to this, where patients’ side effects were considered to become worse over time, as their body started refusing the drugs.

*In comparison to the initial stage of my treatment the difficulties I have now are much easier. I don’t suffer that much… I think my body got used to the drugs*. P07

*Actually after the sixth…after the 7th or 8th month of treatment I realised that my body started refusing*. P33

Some patients perceived treatment as poisoning other parts of the body, helping the lungs at the expense of the liver, kidneys, and stomach, for example. Some felt that their body was being ruined by drug toxicity, feeling unable to continue with treatment as a result, and fearing death from liver failure.

*If you lose liver then you will die soon. If you lose lung you will live a bit longer… I said I wouldn’t take them anymore. I will spoil my liver, my liver will be destroyed*. P27

*It’s like it has made all my bodies not function… all is ruined*. P29

*Now I vomit because I am poisoned with the drugs.* P33

Certain patients expressed a sense of treatment fatigue, feeling that they had had enough of the burden of drug-taking and experiencing side effects each day. This was seen to undermine their motivation to continue with treatment and caused some patients to want a break. It was given as the reason for some stopping treatment or missing doses.

*I am fed up and refused completely. I stopped for 2 months*. P18

Individuals were seen as tolerating drug side effects differently, with some patients not feeling able to tolerate the drugs at all. Certain patients found that tolerance became more difficult as the week progressed, with a perceived build-up effect, highlighting the importance of a rest day from treatment.

*When I took those drugs I suffered. I had been suffering for 2 years, vomiting, I couldn’t tolerate at all. I used to vomit for 2 years, it didn’t stop.* P25

*We don’t take the tablet on Sundays… on those days I feel myself good like old times*. P35

Medical side-effect management had mixed reviews from patients and health practitioners. Some found that anti-side-effect drugs helped, others experienced no difference, and one patient felt worse with anti-emetic drugs. Several patients described feeling reluctant to try anti-side-effect drugs due to an aversion to increasing their pill intake. Health practitioners also described some patients hiding side effects to avoid increased pill burden. This factor limited the impact of medical support for side-effect tolerance and therefore adherence.

*If I take the anti-vomiting drug my drugs neither digest nor come out from my mouth. And I feel very bad. Better not to take anti-vomiting drug but try to keep the drugs for at least half an hour or an hour or so.* P33

A psychological ‘sphere’ to drug tolerance was described by many patients and health practitioners, with mood and mind set reportedly influencing the extent to which side effects were felt and experienced. This links to the concept of patients’ views around self, health, and the effect of the drugs. Focusing on positives was seen to lessen side effects and improve tolerance; whereas being around other patients discussing side effects and focusing on negatives was said to make treatment-taking and tolerance more difficult.

*If you suffer and you think of something bad you will not be able to take the drugs, it’ll be even worse. And if you are joyful and don’t think of anything bad then the drugs will not… [affect] you much*. P11

*These drugs also can work depending on your mood. If a man is in a good mood then these drugs can be absorbed well and there might be no side effects. That’s why after you take the drugs you should….[think] positively* P21

*When people have some hope, when people see life very happy they tolerate more or less better than people who are very depressed.* HP 08

Some patients described using visualisation techniques to build a positive mind set, helping with drug tolerance. These included visualising the drugs helping the body, strengthening it and providing a cure, as well as tactics such as imagining the drugs were grapes or sweets. Conversely, some patients described developing a negative association with drugs, which led to them feeling nauseous at the sight or even thought of them, including drugs not related to MDR-TB.

*I used to vomit when I saw them.* P31

*You try to distract yourself a bit like you are taking only grapes.* P33

Distractions from treatment were referred to as helping with drug tolerance and treatment-taking, which supports the notion of visualisation as a useful technique. Patients described activities such as walking outside, talking to friends or family, watching television, listening to music, or keeping busy in some way. Having distractions like these were said to be important, helping the days and treatment-taking pass more easily and improving mood.

*I come home after taking drugs and then go for a walk. That time you can forget, distract yourself… It helps to be distracted from the drugs*. P19

*If you walk around or chat with someone, if you talk about something good to someone or listen to music then I think the drugs will digest well.* P21

Patients described different tactics to help them digest and tolerate the drugs, which varied for each individual. The coping mechanisms described included splitting doses or taking the drugs in one go, taking them with yoghurt or watermelon, lying down for 2–3 h after taking them, and performing mindfulness and meditation exercises with counsellor support. Food was mentioned as being important for drug tolerance, helping with taking the drugs and masking their taste, as well as reportedly improving side effects. Certain patients also highlighted the importance of a peaceful environment, with quarrelling, nervousness, and anger seen as undermining motivation for drug-taking, as well as worsening drug tolerance and disease progression.

*They say if a man is nervous then the vessels in lungs would be swollen and explode.* P18

## Discussion

We found several factors that influenced the ability to adhere to MDR-TB treatment. Hope and high quality knowledge can support adherence; autonomy and control can enable optimal engagement with treatment-taking; and perceptions of the body, self, treatment, and disease can influence drug tolerance. As far as we are aware, the influence of patient autonomy and control on TB treatment-taking has not previously been described. In particular, we found that the autonomy of some married women around treatment-taking was undermined through their societal position as daughter-in-law, compromising their ability to adhere to treatment. We also found that patients’ engagement with treatment and adherence could be undermined through hierarchical practitioner-patient relationships that displaced authority, ownership, and responsibility from the patient.

The importance of patient knowledge and information for adherence and retention in treatment has been documented [24]. Lack of information is associated with LFT [25] and poor treatment outcomes [26], and patient education is said to be one of the most effective interventions for reducing LFT in DR-TB [5]. However, our finding that patients had insufficient knowledge despite being in a programme that provided pre-treatment information and on-going patient counselling is noteworthy, particularly as some of these patients had been on treatment for considerable periods. Our study adds important additional insight into the role of mind set and visualisation for drug tolerance, building on previous literature on the influence of drug side effects on adherence to MDR-TB treatment [12, 15, 24, 27, 28].

In the first theme that we identified, the presence of TB knowledge was seen to increase the likelihood of continuous engagement with treatment through influences on motivation, belief in the need for treatment and its efficacy, and hope for cure. Several patients, particularly those partially adherent or LFT, appeared to lack understanding about the implications of missing doses and the need to complete the full course of treatment. Certain patients defined themselves as cured once their symptoms improved or their culture results became negative, and thus stopped treatment before completing the full course. Several LFT patients did not believe they had TB despite receiving pre-treatment information and counselling during treatment. Doubt and distrust of the information received was expressed by several patients, exacerbated through changes in culture results and drug sensitivity test analyses. Some patients also appeared to be vulnerable to misinformation and myths, including those relating to alternative treatment options that were deemed to be less arduous and lengthy. Other studies have found that perceptions around cure and belief can cause adherence difficulties, with patients deciding they no longer need treatment once symptoms improve with treatment for MDR-TB [8] and drug-sensitive TB [3]. MDR-TB patients in other settings have been found to fear treatment as harmful and ineffective [8], and to not understand the implications of missing doses [7].

The active approach to information seeking expressed by several patients in our study could indicate insufficient provision of information from official sources within the programme. The patients who expressed actively seeking further information about their disease and treatment were all female, and most were young. Several participants expressed the need for more patient education, available in different formats and at different stages throughout the treatment course. This confirms the need to capitalise on the existing desire of patients for knowledge. Using mechanisms that enhance trust, such as peer-to-peer information could increase the likelihood of patients believing in the treatment. Certain patients also demonstrated a proactive approach to motivation through peer support by self-forming patient support networks. The patients who described valuing peer support were mostly young (under 30 years), male and female, and the majority were adherent. Counsellor and peer support was key to instilling hope for patients, similar to research showing that counsellor support is crucial for increasing patients’ belief in cure [27].

In the second major theme we identified, patients’ autonomy and engagement around their treatment-taking was found to be influenced by social discourse on TB and authoritative relationships in their family, social, and medical lives. These affected patients’ ability to prioritise treatment over conflicting demands, as well as their sense of responsibility and ownership. Patients with resilience, intrinsic motivation, and who valued their health were said by health practitioners to be better able to continue with treatment and overcome its challenges. Treatment for many patients with MDR-TB has an impact on their sense of self and their lives through hindering their ability to work, study, and perform normal activities [27, 28]. In our study, patients’ limited control and autonomy over their lives and TB care influenced their ability to adhere to treatment. Certain female patients asserted the challenges they faced trying to balance the requirements of their role as daughter-in-law with those of treatment. Married female patients whose families did not support treatment often found themselves unable to continue, highlighting the need for involvement of family members where possible, and the availability of other options such as hospital-based care where this is not feasible. Patients reported that their social and financial responsibilities, including the need to work and earn money, could create barriers to treatment-taking. This was particularly mentioned by male patients, who had a responsibility to provide for their families, and some of them felt unable to continue with treatment as a result. These socio-economic barriers to treatment continuation have been noted previously as being associated with LFT or poor adherence [4, 7, 10].

The power inherent in the practitioner-patient relationship could shape patients’ interactions with their treatment. A “good patient, bad patient” dynamic was reflected, with some patients who adhered well reporting praise from doctors and nurses, whereas other patients felt blamed, shouted at, and inspected. This style of communication was seen to deter patients from feeling able to raise and discuss the challenges they were facing, and undermined their control and ownership over their treatment. This in turn led in some cases to a rebellious response from patients with regard to taking treatment, a response which has been explored further in Foucault’s work on the power dynamic and hierarchy within practitioner-patient relationships [29]. Health practitioner training is needed to improve practitioner-patient communication, collaboration, mutual commitment, and decision-making. The importance of adequate heath practitioner training [7] and an approach that enhances patient ownership and involvement [24] are clear.

The third theme we found highlights the relevance of disease embodiment and the ways in which a person perceives and interacts with treatment both physically and psycho-socially. The fact that all patients asserted experiencing side effects at some point in their treatment demonstrates the difficulties associated with taking such toxic drugs. Several patients described perceptions that the drugs were poisoning them, for example curing their lungs at the expense of other organs such as their liver. Other studies have reported patients’ experiences with side effects [12, 27, 28], with one qualitative study describing the negative impact of adverse drug effects on adherence [24]. While other studies have found the number of drugs as increasing risk of LFT [15], our findings provide insight into the ways in which a patient’s outlook can influence MDR-TB drug tolerance.

Patients felt better able to tolerate the treatment if they had a more positive view of the drugs. Using techniques such as visualisation and distractions from treatment reportedly eased drug tolerance and therefore promoted drug-taking. Patients reported feeling drug side effects more acutely if they focused on them, or were around other patients suffering from them. At the same time, reinforcement of health care workers’ skills to support patients with long-term treatment and drug intake could help patients to believe in the chance of achieving a successful treatment outcome, which in turn could motivate good adherence to treatment.

### Limitations

While identification of participants for potential recruitment in the study followed several steps, the role of health practitioners in approaching potential participants, informing them about the study and requesting their voluntary participation may have influenced recruitment in its reliance on their relationship with these individuals. However, as these health practitioners were predominantly counsellors who had supportive relationships with selected patients this should not have negatively influenced recruitment and may have encouraged participant openness. LFT participants took longer to identify and required adaptation of the recruitment approach to include direct introduction of the study by the Principal Investigator (PI). As we were unable to include patients who were abroad, this hindered exploration of their experiences with stopping treatment early. However, the financial and social responsibilities that can influence patients’ adherence have been portrayed, which may also have applied to these patients (some of whom likely migrated for employment reasons).

It is acknowledged that participants could have shared a narrative that portrayed the account they thought the researcher wanted to hear; for example, health practitioner criticism was more directly described by practitioner participants than by patients, which could indicate an association of the researcher with the health programme. However, the fact that the researcher was not from the programme potentially countered this effect; participants were generally open regarding their experiences, views, and struggles. The role of the PI in shaping the data must also be acknowledged and reflected upon. While steps were taken to reduce researcher bias during the data collection and analysis process, for example through the review of coding and analysis by a second researcher, the PI’s involvement in designing the study, and in data collection and analysis, is likely to have influenced the study findings. As is to be expected in qualitative research, the concepts arising in this study may be generalisable to the larger population treated in the programme, but these may not be found in other contexts or in dissimilar settings.

## Conclusions

Patients experienced a variety of challenges throughout their treatment, reinforcing the need for an individualised and holistic approach to adherence support. This study suggests that quality knowledge, hope and belief, and patient autonomy and control can support MDR-TB treatment-taking; and that there is a psychological sphere to treatment which influences drug tolerance.

Improving patients’ knowledge and understanding relating to TB treatment and disease could help them have the belief, hope, determination, and motivation to complete treatment. A space within which patients have a sense of authority and autonomy over their treatment must be created, for example through enhanced practitioner-patient communication and minimising social barriers, so that patients are active participants in their care and feel ownership and responsibility for their treatment. Finally, while adverse drug effects are experienced by the majority of patients, mechanisms have been found to help patients cope with and overcome difficulties with them and should be implemented as part of adherence strategies, such as the employment of visualisation techniques and distractions from treatment, which were found to ease the burden of side effects in this study.

As we move towards the introduction of new drugs and treatment regimens for MDR-TB, the potential impact on adherence and how best patients can be supported should be considered as integral components of future studies and programmes.

**Table:** 

Policy and Practice Recommendations: towards enhancing the patient-centred approach	
Patient knowledge, understanding and hope	Increase the availability of information to patients at different stages of the treatment course (beyond point of diagnosis) Patient information should also include a range of formats, including peer-to-peer and interactive Increase community-level sensitisation on MDR-TB Expand peer support services for MDR-TB patients Counsellor support should be made available to patients, tailored to meet individual psychosocial support needs, including the option for family engagement and to support gendered experiences which can influence treatment-taking
Patient autonomy and control over treatment-taking	Practitioner-patient relationships should facilitate dialogue so patients feel able to discuss any challenges, concerns and queries they might have Offer practitioner training and engagement on communication Offer support for practitioners who may face challenges with the burden of workload and responsibility for achieving successful patient outcomes
Patient perceptions of the self, body, treatment and disease on tolerance – drug side effects	Offer support for patients around mind set and treatment-taking – as focusing on positives and seeing treatment as strength-giving was found to offer the potential to ease difficult drug side effects Employment of visualisation techniques and distractions from treatment could also support patients with drug tolerance

## Abbreviations

DOT, directly observed therapy; HP, health practitioner; LFT, loss from treatment; MDR-TB, multi-drug resistant tuberculosis; MoH, Ministry of Health; MSF, Médecins Sans Frontières; PI, principle investigator
